# Environmental filtering drives the establishment of the distinctive rhizosphere, bulk, and root nodule bacterial communities of *Sophora davidii* in hilly and gully regions of the Loess Plateau of China

**DOI:** 10.3389/fmicb.2022.945127

**Published:** 2022-07-22

**Authors:** Li Jing, Ai Jia-min, Liu Xiao-dong, Jiang Ying-ying, Zheng Chao-chao, Zhao Rui-hua, Deng Zhen-shan

**Affiliations:** College of Life Sciences, Yan’an University, Yan’an, China

**Keywords:** *Sophora davidii*, root nodules, high-throughput sequencing, diversity, biogeographic patterns, network pattern

## Abstract

In addition to the rhizobia, other non-rhizobial endophytes (NREs) have been simultaneously isolated from the root nodules. The existence of NREs in leguminous root nodules is a universal phenomenon, and they have the potential to enhance legume survival, especially under conditions of environmental stress. However, the diversity and biogeographic patterns of microbial communities inhabiting root nodules are not well studied or understood. Here, we explored and characterized the diversity of NRE bacteria by using 16S rRNA gene high-throughput amplicon sequencing. Additionally, we compared the biogeography and co-occurrence patterns in review of the bacterial microbiota inhabiting the rhizosphere, the bulk soil and the root nodule bacterial communities associated with *Sophora davidii*, a native N-fixing wild leguminous shrub in hilly and gully regions of the Loess Plateau of China. The results showed the presence of a large diversity of bacteria belonging to 81 phyla, 154 classes, 333 orders, 463 families, and 732 genera inside the nodules. Proteobacteria were dominant in the nodule and rhizosphere soil samples, and Actinomycetes were dominant in the bulk soil samples. *Mesorhizobium* was the dominant genus in the nodules, accounting for between 60.15 and 83.74% of the bacteria. The microbial community composition of the NRE in the root nodules differed from that in the rhizosphere soil and the bulk soil of *S*. *davidii*. Moreover, we found that the biogeographic patterns and assembly process of the rhizobia and non-rhizobia communities differed in the root nodule, the rhizosphere soil and the bulk soil. Furthermore, the correlation analysis between the soil’s physical and chemical properties and the bacteria showed that available phosphorus was the predominant factor affecting the bacterial diversity within the rhizosphere soil. Finally, our results revealed that the microbial network diagram of co-occurrence patterns showed more complexes in the soil than in the root nodules. This indicates that only specific microorganisms could colonize and thrive in the rhizosphere through the selection and filtering effects of roots. In conclusion, there are significant differences in bacterial community composition in the nodules, rhizosphere and bulk soil in the hilly and gully region of the Loess Plateau, which is the result of environmental filtration. Our study improves the understanding of the biogeographic patterns and diversity of bacterial microbiota inhabiting root nodules and can help quantify and define the root nodule assemblage process of *S*. *davidii*.

## Introduction

One of the unique characteristics of legumes is that they can form special root or stem nodules on plant roots. The legume root nodule is a unique niche for symbiotic nitrogen-fixing occupied by bacteria so called rhizobia. In recent years, many studies on legumes have found that root nodules are a complex niche, in addition to rhizobium, there have also been non-rhizobial root nodule endophytes (NRE) isolated from legume nodules without clear indications of their role within the host, and an absence of positive nodulation tests (De Meyer et al., 2015). The reported NREs include *Agrobacterium* (Beiierinck and van Delden, 1902; Cummings et al., 2009), *Bacillus* (Rajendran et al., 2008; Palaniappan et al., 2010), *Pseudomonas* (Aserse et al., 2013), *Enterobacter* (Benhizia et al., 2004; Ibáñez et al., 2008), *Paenibacillus* (Zakhia et al., 2006; Deng et al., 2011; Lai et al., 2015), *Burkholderia* (Raupach and Kloepper, 1998; Rasolomampianina et al., 2005; Diouf et al., 2007), *Staphylococcus* (Deng et al., 2011), *Pantoea* (Li et al., 2007; Aserse et al., 2013), *Methylobacterium* (Sy et al., 2001; Palaniappan et al., 2010), *Herbaspirillum* (Valverde et al., 2003; Weiss et al., 2012), *Microvirga* (Ardley et al., 2012), *Paraburkholderia* (Sawana et al., 2014), *Rhodococcus* (Ampomah and Huss-Danell, 2011), *Variovorax* (Hoque et al., 2011; Aserse et al., 2013), *Klebsiella* (Pandya et al., 2013), *Arthrobacter* (Barnawal et al., 2014), *Brevibacterium* (Xu et al., 2014; Gopalakrishnan et al., 2016), and *Streptomyces* (Schrey and Tarkka, 2008; Sreevidya et al., 2016). Although different bacteria have been isolated from root nodules of various leguminous plants, there are still many bacteria that cannot be obtained by pure culture, therefore, the biological significance of these associations is largely unknown.

Although many of the NREs are not capable of nitrogen fixation, they have the potential to enhance legume survival, especially under conditions of environmental stress (Martínez-Hidalgo and Hirsch, 2017). And they are often detected within nodules obtained from soil.

These bacteria are opportunistic since they can infect nodules when rhizobia induce nodule formation (Ibáñez et al., 2008; Leite et al., 2017; Sarah and Djamel, 2021), and more than one species of non-rhizobia could be found in the nodules of *Trifolium repens* (Olenska et al., 2020). As many of the taxa identified in nodules are common in soil, it is possible that many of them are inadvertently entrapped during the rhizobia nodulation process. This provides evidence that both the soil bacterial community and the host plant might influence the nodule microbiome. Zgadzaj et al. (2015, 2016) concluded that endophyte nodule occupancy is host-controlled and that the ability of legumes to select a broad taxonomic range of root-associated bacteria along with rhizobia is likely to contribute to plant growth and ecological performance. However, Leite et al. (2017) reported that the non-rhizobial bacterial community in cowpea nodules was influenced by the soil type rather than the plant genotype.

However, there is currently limited information regarding the entry mode of endophytic bacteria, and the biological significance of these non-rhizobial bacteria is largely unknown. In addition to the plants themselves, rhizosphere soils are another niche of root-affected soil, and they represent a special area affected by plant roots (Li et al., 2021). The microbial community structure within the rhizosphere soil also has an important relationship with plant growth and development. Therefore, the simultaneous study of the diversity of endophytic bacteria and rhizosphere bacteria is of great significance for elucidating the function of these bacteria and tapping these biological resources (Tian and Zhang, 2017). Biogeographic patterns of microorganisms play important roles in generating and maintaining biodiversity, and community assembly processes are usually studied simultaneously with microbial biogeography (Zhang K. et al., 2017; Liu et al., 2021). Co-occurrence model analysis was applied to explore the potential interactions within niche spaces shared by community members (Kara et al., 2013).

*Sophora davidii* (Franch.) Skeels is a perennial shrub of the leguminous family, it grows in river valley dunes and bushes on hillside roads below 2,500 m. It is a native plant in China and is widely distributed in Guizhou, Ningxia, Shaanxi, Yunnan, and Sichuan provinces (Tai et al., 2011). As a deep-rooted nitrogen-fixing wild leguminous shrub, *S*. *davidii* has an important ecological value in plant community succession, soil improvement and soil erosion control in arid areas due to their strong adaptability, strong stress resistance, and developed root system (Wu et al., 2007). Moreover, compared with herbaceous plants, nitrogen-fixing shrubs can significantly increase the contents of available nitrogen and phosphorus in soil (Song et al., 2010). At present, research on *S*. *davidii* is mainly focused on the analysis of their chemical constituents (Ma et al., 2021; Zhang et al., 2021). A recent study focused on the diversity and distribution of rhizobia associated with *S*. *davidii* (Cao et al., 2021). However, studies on non-rhizobial composition and assembly in root nodules have not been reported, and little is known about the biodiversity of endophytic bacteria residing within the roots of *S*. *davidii.*

In this study, we compared the biogeography and co-occurrence patterns of the bacterial microbiota inhabiting the rhizosphere, the bulk soil and the root nodule bacterial communities of *S*. *davidii* collected from the hilly and gully regions of the Loess Plateau of China. The aim was to address the following questions: (i) clarify the diversity and community structure of rhizobia and the NREs of *S*. *davidii* and to estimate the ecological drivers for the bacterial communities in the rhizosphere, the bulk soil and the root nodule; (ii) clarify the distinct bacterial microbial co-occurrence patterns of the three compartments; and (iii) identify the biogeographic patterns and assembly process of the rhizobia and non-rhizobia communities that vary among the compartments.

## Materials and methods

### Sampling sites and nodule collection

**FIGURE 1:**
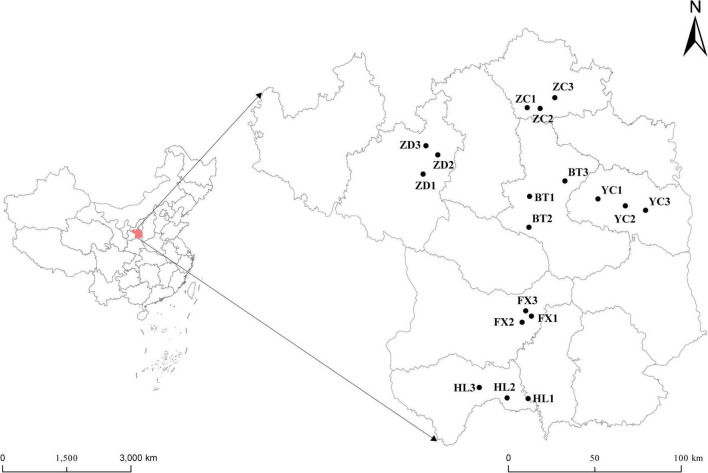
Geographical distribution of the six sampling sites of *S*. *davidii* in China. The simplified map shows six sampling areas of Huangling (HL), Fuxian (FX), Baota (BT), Yanchang (YC), Zhidan (ZD), and Zichang (ZC) in Yan’an city, Shaanxi Province. Each sampling area includes three sampling points.

Root nodules of the *S*. *davidii* at flowering-fruiting stages were collected from May to July 2021 at six sample sites in the hilly and gully regions of the Loess Plateau of China. The geographical locations of the sampling sites are shown in Figure 1 (the corresponding geographical features are shown in Supplementary Table 1). The distance between sampling sites was at least 10 km. Each sampling site included three subsites separated by more than 1 km. Fifteen plants with a spacing of at least 30 m were randomly selected and uprooted from each subsite. Three to four undamaged and healthy root nodules of similar size were excised from the lateral roots of each plant. These nodules were brushed away from the soil debris and then immediately desiccated over silica gel in 1.5 mL microtubes (Vincent, 1970). At the same time the root nodules were collected, the rhizosphere soil and bulk soil were collected. Rhizosphere soil is collected by digging the entire plant root out of the soil with minimum injury to the roots, gently shaking the roots until the soil that is not tightly adhering to the roots is removed, and then vigorously shaking the roots to collect the soil tightly adhering to the roots into sterile bags. The soil outside the rooting area is collected as bulk soil (Wang and Zabowski, 1998). One part of the soil samples were air-dried in the shade. The soils samples physicochemical characteristics; organic matter (SOM), total nitrogen (TN), ammonium nitrogen, nitrate-nitrogen (NN), alkali hydrolysable nitrogen, available phosphorus (AP), available potassium (AK), pH, and electrical conductivity (EC) were measured using the protocols described by the United States Department of Agriculture [USDA] (1996). The remainder was stored at –80^°^C for high-throughput sequencing at Novogene Biotechnology Co., Ltd. (Tianjin, China).

To eliminate other microbial interference on the nodules’ surface, a surface sterilization procedure was performed on the nodules. The healthy and unruptured root nodules were rinsed under running water, wiped dry with filter paper, surface sterilized by immersion in 95% ethanol for 30 s, rinsed with sterile distilled water three times, then immersed in 5% sodium hypochlorite for 3 min, and rinsed with sterile distilled water eight times. To verify the effect of surface sterilization, the sterile distilled water washed for the last time was added to potato dextrose agar plates. If there was no colony growth on the plate, it indicated that the surface disinfection of the root nodules was effective. All samples were stored at –80^°^C until DNA extraction.

### DNA extraction and sequencing

Approximately 2 *g* of disinfected root nodules was put into a precooled mortar, and then liquid nitrogen was added and thoroughly ground into a fine powder. Total DNA was extracted using a high-efficiency Plant Genomic DNA Extraction Kit (Tiangen, DP350, China). Soil samples (0.2 *g*) were weighed, and a soil genomic DNA extraction kit (Tiangen, DP336, China) was used to extract the soil DNA. The DNA concentration and purity were detected and evaluated by a NanoDrop 2000 spectrophotometer (Thermo Scientific, Waltham, MA, United States) and 1% agarose gel electrophoresis. The samples were diluted with sterile distilled water to 1 ng/μL. Primers 16S-341F (5′-CCTAYGGGRBGCASCAG-3′) and 16S-806R (5′-GGACTACNNGGGTATCTAAT-3′) were used for the PCR amplification of the 16S rRNA V3+V4 region. PCRs were carried out with 15 μL of Phusion^®^ High-Fidelity PCR Master Mix (New England Biolabs), 0.2 μM forward and reverse primers and approximately 10 ng of template DNA. Thermal cycling consisted of initial denaturation at 98^°^C for 1 min, followed by 30 cycles of denaturation at 98^°^C for 10 s, annealing at 50^°^C for 30 s, and elongation at 72^°^C for 30 s. Finally, the samples were incubated at 72^°^C for 5 min. The same volume of 1 × loading buffer (containing SYBR green) was mixed with the PCR products, and electrophoresis was performed on a 2% agarose gel for detection. The PCR products were mixed in equidensity ratios. Then, the PCR products were purified with a Qiagen Gel Extraction Kit (Qiagen, Germany).

Sequencing libraries were generated using a TruSeq^®^ DNA PCR-Free Sample Preparation Kit (Illumina, United States). The library quality was assessed on the Qubit@2.0 Fluorometer (Thermo Scientific) and Agilent Bioanalyzer 2100 system. Finally, the library was sequenced on an Illumina NovaSeq 6000 platform, and 250 bp paired-end reads were generated (Zhang C. S. et al., 2017).

### Microbial community analysis

The paired-end reads were assigned to samples based on their unique barcode and truncated by cutting off the barcode and primer sequence. Then, the paired-end reads were merged using FLASH (VI.2.7)^1^ (Magoc and Salzberg, 2011), a very fast and accurate analysis tool that merges paired-end reads when some of the reads overlap the read generated from the opposite end of the same DNA fragment. The splicing sequences were assigned raw tags. The quality filtering of the raw tags was performed under specific filtering conditions to obtain a high-quality clean tag (Bokulich et al., 2013) according to the QIIME (Version 1.9.1)^2^ (Caporaso et al., 2010) quality control process. The tags were compared with the reference database (Silva database)^3^ using the UCHIME algorithm (UCHIME Algorithm)^4^ (Edgar et al., 2011) to detect chimera sequences, and then the chimera sequences were removed (Haas et al., 2011). Then, the Effective Tags were finally obtained.

Sequence analysis was performed by UPARSE software (UPARSE v7.0.1001)^5^ (Edgar, 2013). The operational taxonomic units (OTUs) were clustered according to 97% similarity (Edgar, 2010) and assigned to each sample with the QIIME pipeline for sequence analysis (Caporaso et al., 2010).

Representative sequences for each OTU were screened for further annotation. For each representative sequence, the Silva Database (see text footnote 3; Quast et al., 2013) was used based on the Mothur algorithm to annotate taxonomic information. To study the phylogenetic relationship of different OTUs and the difference in the dominant species in different samples, multiple sequence alignments were conducted using MUSCLE software (Version 3.8.31)^6^ (Edgar, 2004).

### Network construction

To determine the co-occurrence network pattern of the bacterial communities in root chambers and the structural differences of bacterial communities in different niches, Spearman correlation analysis was carried out among the most abundant bacterial genera in each root chamber. The data with a correlation coefficient greater than 0.6 and a *p*-value < 0.5 (Barberán et al., 2012) were selected to form a correlation network through pairwise comparison of genus abundance, in which each node represents a genus and each edge represents a significant correlation between the two nodes. The number of nodes and edges, average path length (APL), network diameter, average degree, graph density, clustering coefficient (CC), and modularity were calculated by igraph packages in R language, and the results were visualized by Gephi software (Bastian et al., 2009).

### Statistical analysis

One-way ANOVAs and the Tukey–Kramer test were used to test the alpha diversity of the samples (including observed species, Chao l, Shannon, Simpson, ACE and Good coverage). Beta diversity [including principal coordinate analysis (PCoA) and unweighted pair-group method with arithmetic means (UPGMA)] was used to analyze and evaluate the differences in species complexity in the samples. A least discriminant analysis (LDA) effect size (LEfSe) taxonomic cladogram was used to identify bacterial biomarkers (LDA > 4) in the different treatments using LEfSe software (Segata et al., 2011). Alpha diversity and beta diversity were calculated by QIIME and displayed with R software (Caporaso et al., 2010). The correlation between the soil’s physicochemical properties and the bacterial community was calculated and plotted by R.

## Results

### Physicochemical characteristics of the soil

The physicochemical characteristics of the rhizosphere soil of *S*. *davidii* are shown in Table 1. In general, the soil of each sampling site was alkaline, and the pH was between 7.85 and 8.26. The contents of AP and TN in the sample ZCS were 78.87 and 53.93 mg/kg, respectively, which were significantly higher than those of other samples. The content of AK in the sample FXS was the highest at 249.33 mg/kg. The range of soil organic matter was between 5.25 and 25.24 g/kg. The sample HLS had an organic matter content of 25.24 g/kg, which was significantly higher than that of the other samples. The contents of AP, AK and organic matter in the rhizosphere soil were generally higher than those in the bulk soil. The contents of NN and alkaline hydrolyzable nitrogen in the rhizosphere soil at different sample sites were lower than those in the corresponding bulk soil, but the content of NN was the inverse. In the Huangling and Fuxian samples, the content of TN in the rhizosphere soil was lower than that in the bulk soil, but the results were inverse in the other four sample groups. The conductivity of the sample ZDS, which was 681.67 was much higher than that of the other samples.

**TABLE 1:** Soil physicochemical property of each sample.

Sample name	Available phosphorus (AP) (mg/kg)	Available potassium (AK) (mg/kg)	Organic matter (SOM) (g/kg)	Nitrate nitrogen (NN) (mg/kg)	Ammonium nitrogen (AMN) (mg/kg)	Alkaline hydrolyzable nitrogen (AHN) (mg/kg)	Total nitrogen (TN) (mg/kg)	pH	Electrical conductivity (EC)
HLS	16.19 ± 5.87d	199.00 ± 21.93b	25.24 ± 1.24a	6.62 ± 0.54cd	5.24 ± 0.44a	15.77 ± 0.23bcd	40.20 ± 4.07abc	7.92 ± 1.00de	187.17 ± 22.37c
HLCK	8.10 ± 1.08d	81.67 ± 11.72fg	7.36 ± 0.48de	15.73 ± 2.66a	4.09 ± 0.76abc	27.37 ± 2.93a	51.84 ± 12.43ab	8.19 ± 0.12abc	133.03 ± 4.45c
FXS	14.23 ± 0.89d	249.33 ± 28.10a	10.23 ± 1.96d	4.94 ± 0.64cd	5.04 ± 1.02abc	12.97 ± 4.70bcd	30.09 ± 10.01bc	7.86 ± 0.10e	250.63 ± 96.54b
FXCK	5.25 ± 1.07d	111.67 ± 14.19ef	5.25 ± 0.23e	11.54 ± 2.78b	2.91 ± 0.21abc	18.20 ± 3.38b	34.31 ± 8.17abc	8.05 ± 0.17cd	159.00 ± 20.97c
BTS	16.05 ± 6.81d	152.67 ± 51.01cd	15.59 ± 2.53bc	3.28 ± 0.35d	4.91 ± 1.18abc	10.27 ± 3.53cd	40.70 ± 13.95abc	8.09 ± 0.04abcd	159.33 ± 11.87c
BTCK	10.00 ± 4.77d	81.00 ± 16.37fg	9.24 ± 1.00d	6.61 ± 2.04cd	3.33 ± 0.09ab	12.73 ± 2.01bcd	26.61 ± 3.92c	8.16 ± 0.03abc	147.83 ± 3.31c
YCS	19.42 ± 5.02d	125.67 ± 4.73de	14.34 ± 4.63bc	4.60 ± 0.65d	5.09 ± 3.58abc	9.33 ± 3.04d	43.87 ± 22.70abc	8.06 ± 0.08bcd	180.70 ± 18.56c
YCCK	8.45 ± 3.19d	58.33 ± 11.68g	7.39 ± 0.55de	8.27 ± 3.51c	3.14 ± 0.88abc	17.13 ± 5.15bc	42.65 ± 8.87abc	8.20 ± 0.05abc	122.60 ± 38.81c
ZDS	57.81 ± 3.71b	172.67 ± 29.87bc	17.76 ± 2.41b	3.41 ± 0.48d	4.03 ± 0.90abc	10.93 ± 4.30cd	49.60 ± 4.54ab	7.96 ± 0.11de	681.67 ± 18.77a
ZDCK	20.30 ± 7.82d	88.00 ± 8.19efg	7.52 ± 1.97de	6.77 ± 2.49cd	2.56 ± 0.86c	14.00 ± 3.40bcd	33.65 ± 0.60abc	7.85 ± 0.12e	156.83 ± 18.48c
ZCS	78.87 ± 24.89a	167.00 ± 22.52bc	13.93 ± 1.68c	4.33 ± 0.95d	4.53 ± 0.56abc	10.33 ± 4.96cd	53.93 ± 15.44a	8.23 ± 0.07ab	162.43 ± 5.76c
ZCCK	41.14 ± 8.72c	81.33 ± 12.66fg	8.92 ± 1.09de	5.82 ± 1.41cd	2.62 ± 0.82bc	18.83 ± 3.70b	31.55 ± 9.62bc	8.26 ± 0.03a	126.23 ± 8.91c

HLS, FXS, BTS, YCS, ZDS, and ZCS indicates the rhizosphere soil samples of the six sampling sites, respectively. HLCK, FXCK, BTCK, YCCK, ZDCK, and ZCCK indicates the bulk control soil samples of the six sampling sites, respectively. N = 3. These data represent mean ± standard deviation. Different lowercase letters indicate significant differences in this parameter between sites. The mean value was compared by one-way ANOVA (P < 0.05).

### Microbial composition and diversity in the root nodule, the rhizosphere, and the bulk soil

**FIGURE 2:**
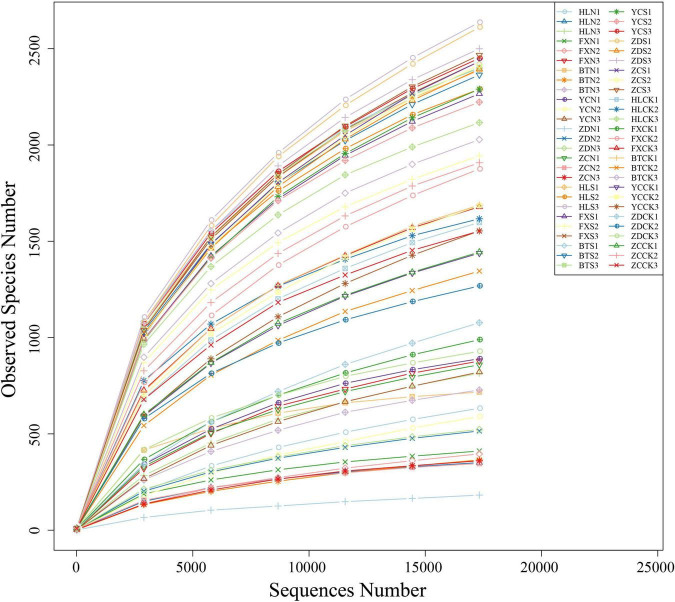
Rarefaction curves based on the sequences of the V3+V4 region of the 16S rRNA gene from samples associated with *S*. *davidii*. HLN, FXN, BTN, YCN, ZDN, and ZCN indicate root nodule samples from the six sampling sites. HLS, FXS, BTS, YCS, ZDS, and ZCS indicate the rhizosphere soil samples of the six sampling sites. HLCK, FXCK, BTCK, YCCK, ZDCK, and ZCCK indicate the bulk soil samples of the six sampling sites. *N* = 3.

Illumina MiSeq sequencing generated a total of 4,158,024 raw tags representing 54 samples, with individual reads ranging from 55,931 to 89,355 for each sample. After quality control, a total of 4,130,398 clean tags remained. After removing the chimeras, 3,217,738 effective tags (with an average length of 405–419 bp) were obtained for the generation of OTUs. The values of Q20 ranged from 97.92 to 98.5, indicating that the quality of the databases was high (Table 2). The trend of rarefaction curves suggested that the sequencing data were sufficient for each sample to represent the bacterial community (Figure 2). A total of 114,808 OTUs were detected.

**TABLE 2:** Quantitative statistics of bacterial OTUs and tags of each sample.

Sample name	Raw tags	Clean tags	Effective tags	Base (nt)	Average length (nt)	Q20	GC%	Effective%	OTUs
HLN1	81,795	81,212	62,736	25,443,788	406	98.28	55.18	75.55	902
HLN2	83,563	82,942	63,957	25,950,414	406	98.39	55.17	75.17	421
HLN3	83,489	82,941	65,126	26,451,927	406	98.5	55.16	76.99	1,289
FXN1	80,763	80,155	62,861	25,666,720	408	98.19	54.86	76.62	475
FXN2	86,671	86,333	66,786	27,060,648	405	98.29	55.04	76.97	731
FXN3	79,924	79,441	62,466	25,384,570	406	98.43	55.21	76.87	1,118
BTN1	80,058	79,521	63,454	26,280,276	414	98.09	54.62	78.62	852
BTN2	89,355	88,900	68,539	27,789,650	405	98.42	55.11	76.5	477
BTN3	80,273	79,827	62,050	25,184,716	406	98.45	55.29	76.74	950
YCN1	85,171	84,633	66,256	26,924,293	406	98.47	55.31	76.82	1,143
YCN2	80,753	80,285	60,998	24,736,621	406	98.47	55.07	74.61	1,006
YCN3	77,378	76,944	60,277	24,495,377	406	98.46	55.15	77.37	1,141
ZDN1	79,061	78,603	62,530	25,340,652	405	98.43	55.12	78.76	258
ZDN2	80,029	79,425	62,205	25,334,470	407	98.29	55.09	76.65	655
ZDN3	87,367	86,874	68,399	27,776,200	406	98.4	55.26	78.18	870
ZCN1	89,173	88,722	67,803	27,538,395	406	98.47	55.29	75.56	1,109
ZCN2	87,160	86,568	65,784	26,833,455	408	98.32	54.73	74.63	461
ZCN3	85,796	85,369	68,384	27,750,805	406	98.35	55.37	79.46	466
HLS1	60,408	59,970	45,682	19,077,117	418	98.17	57.03	74.09	3,211
HLS2	61,023	60,638	45,801	19,146,142	418	98.14	56.82	74.49	3,078
HLS3	74,297	73,838	57,328	23,993,016	419	98.02	56.5	76.52	3,600
FXS1	65,492	64,922	49,287	20,617,791	418	98.03	56.95	73.48	3,103
FXS2	79,511	78,969	63,333	26,462,827	418	98.12	56.26	79.5	2,815
FXS3	67,117	66,725	52,107	21,749,843	417	98.1	56.72	77.37	3,378
BTS1	59,946	59,600	46,376	19,355,691	417	98.2	56.34	77.03	3,186
BTS2	64,116	63,626	47,719	19,969,477	418	98.2	57.31	73.23	3,211
BTS3	58,832	58,500	46,345	19,340,325	417	98.19	57.12	78.48	3,273
YCS1	66,485	65,980	49,861	20,826,019	418	98.01	57.36	74.2	3,125
YCS2	55,931	55,559	44,341	18,443,601	416	98.11	56.66	78.57	2,995
YCS3	84,472	83,867	65,564	27,327,985	417	98.11	56.63	77.48	3,482
ZDS1	73,321	72,713	56,729	23,722,954	418	98.07	55.45	76.16	3,576
ZDS2	66,616	66,122	51,902	21,572,625	416	98.09	56.38	77.28	3,248
ZDS3	79,201	78,516	60,441	25,156,208	416	98.01	55.99	75.54	3,643
ZCS1	71,871	71,296	55,814	23,236,450	416	98.07	56.63	77.05	3,332
ZCS2	70,440	69,972	55,450	23,152,838	418	98.03	56.88	78.52	3,277
ZCS3	75,604	75,025	59,651	24,859,493	417	97.97	56.41	78.2	3,589
HLCK1	82,911	82,498	64,701	26,684,844	412	98.23	56.07	77.91	2,401
HLCK2	79,590	78,999	62,135	25,822,080	416	98.02	57.25	77.5	2,420
HLCK3	58,914	58,431	45,271	18,828,754	416	97.92	57.55	75	2,782
FXCK1	77,354	76,842	58,782	24,297,964	413	98.01	57.38	75.63	1,653
FXCK2	76,751	76,304	58,910	24,551,159	417	98.15	55.09	76.66	2,652
FXCK3	85,864	85,342	67,464	28,080,546	416	98.15	54.35	78.53	2,493
BTCK1	75,047	74,665	58,281	24,140,847	414	98.23	56.25	77.05	2,386
BTCK2	78,615	78,146	61,655	25,362,654	411	98.31	54.87	78.02	1,940
BTCK3	73,786	73,235	55,743	23,231,336	417	98.18	57.88	74.09	2,720
YCCK1	84,324	83,782	65,238	27,001,132	414	98.15	56.19	76.97	2,310
YCCK2	72,169	71,556	53,602	22,239,983	415	97.94	57.04	72.76	2,430
YCCK3	84,322	83,774	66,659	27,523,144	413	98.18	56.74	78.78	2,542
ZDCK1	86,130	85,619	68,716	28,297,602	412	98.18	56.59	79.73	1,959
ZDCK2	78,470	77,796	60,108	24,845,499	413	98.16	56.39	75.35	1,719
ZDCK3	84,421	83,814	66,541	27,304,696	410	98.11	57.25	77.51	1,397
ZCCK1	79,197	78,635	61,730	25,564,655	414	98.02	55.53	77.72	2,294
ZCCK2	87,138	86,402	66,068	27,497,939	416	98.07	56.34	74.91	2,837
ZCCK3	80,559	80,025	61,792	25,386,746	411	98.12	55.27	76.49	2,427

**FIGURE 3:**
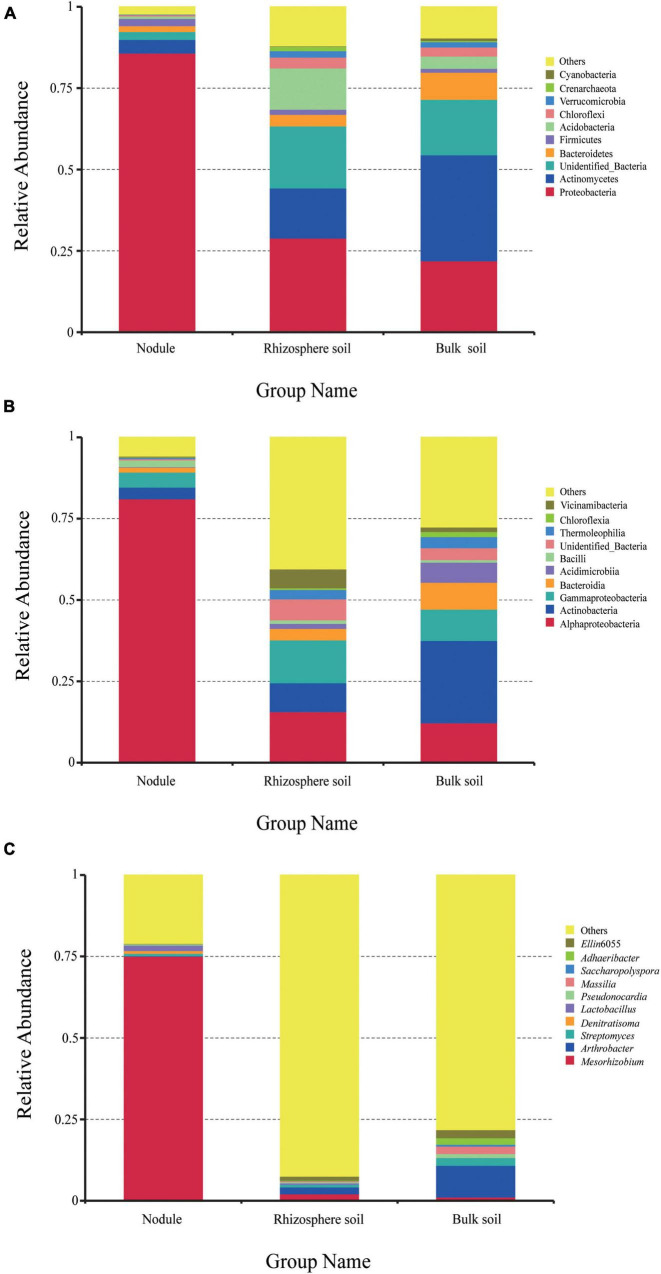
The relative abundance of endophytic bacteria in the three groups of samples at different taxonomic levels. **(A)** is the relative abundance of bacteria at the phylum level. **(B)** is the relative abundance of bacteria at the class level. **(C)** is the relative abundance of bacteria at the genus level.

High-throughput sequencing revealed bacterial community diversity at the phylum level in different samples (Figure 3A). A total of 81 phyla were identified, and the relative abundances of the first 10 phyla are shown in Figure 3A. Proteobacteria had an absolute advantage in root nodules, accounting for 85.77%. Proteobacteria, Actinobacteria and Acidobacteria were the dominant phyla in the rhizosphere soil, accounting for 28.93, 15.41, and 12.71%, respectively. Actinomycetes and Proteobacteria were the dominant phyla in the bulk soil samples, accounting for 32.61 and 21.93%, respectively. The proportion of Acidobacteria in the rhizosphere soil (12.71%) was significantly higher than that in the nodule soil (0.74%) and the bulk soil (3.76%). Compared with the rhizosphere soil and the root nodules, Bacteroidetes were more dominant in the bulk soil (8.30%). However, there was a certain proportion of unrecognized sequences in all of the samples (same below).

At the class level (Figure 3B), Alphaproteobacteria was the most dominant in the root nodules, accounting for 81.13%. In addition, Actinobacteria, Gammaproteobacteria, Bacteroidia, and Bacilli accounted for 3.57, 4.63, 1.5, and 2.06%, respectively, and were the dominant class of bacteria (≥1%). In the rhizosphere soil samples, Alphaproteobacteria, Gammaproteobacteria, and Actinobacteria accounted for a large proportion, at 15.69, 13.19, and 8.88%, respectively. In the bulk soil, Actinobacteria (25.27%), Alphaproteobacteria (12.29%), Gammaproteobacteria (9.64%), Bacteroidia (8.25%), Acidimicrobia (6.19%), Thermoleophilia (3.41%), Chloroflexia (1.56%), and Vicinamibacteria (1.4%) were the dominant bacteria. Overall, the content of Alphaproteobacteria in the root nodules was significantly higher than that in the rhizosphere soil and the bulk soil, while the content of Actinobacteria and Gammaproteobacteria was significantly lower than that in the rhizosphere soil and the bulk soil. It can be seen that different plant-related tissues are selective for the microorganisms present in them.

A total of 732 different genera were detected at the genus level. There were significant differences in the overall composition of bacteria in the different samples. The most prominent phenomenon was that *Mesorhizobium* was overwhelmingly dominant in the root nodules (75.07%) compared with the rhizosphere soil and the bulk soil. In addition to the 10 genera listed in Figure 3C, other strains accounted for 92.45% in the rhizosphere soil and 78.15% in the bulk soil. The results showed that the bacterial communities in the root nodules and rhizosphere soil were diverse, but the bacterial species richness in the root nodules was lower than that in the soil samples.

**FIGURE 4:**
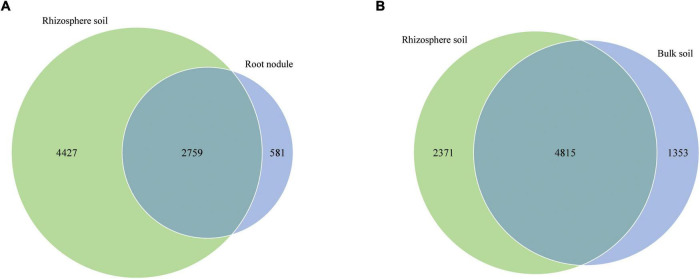
Venn diagrams show the number of shared and unique OTUs in different nodules and soil samples. **(A)** is shared and unique OTUs between nodules and rhizosphere soils. **(B)** is shared and unique OTUs between rhizosphere soil and bulk soil.

In addition, we compared the corresponding number of OTUs in different grouping samples (Figure 4). The number of specific OTUs in nodule and rhizosphere soil samples was 581 and 4,427, respectively, and there were 2,759 common OTUs in total (Figure 4A). The special OTUs of the rhizosphere soil and the bulk soil samples were 2,371 and 1,353, respectively, and there were 4,815 common OTUs in total (Figure 4B).

**FIGURE 5:**
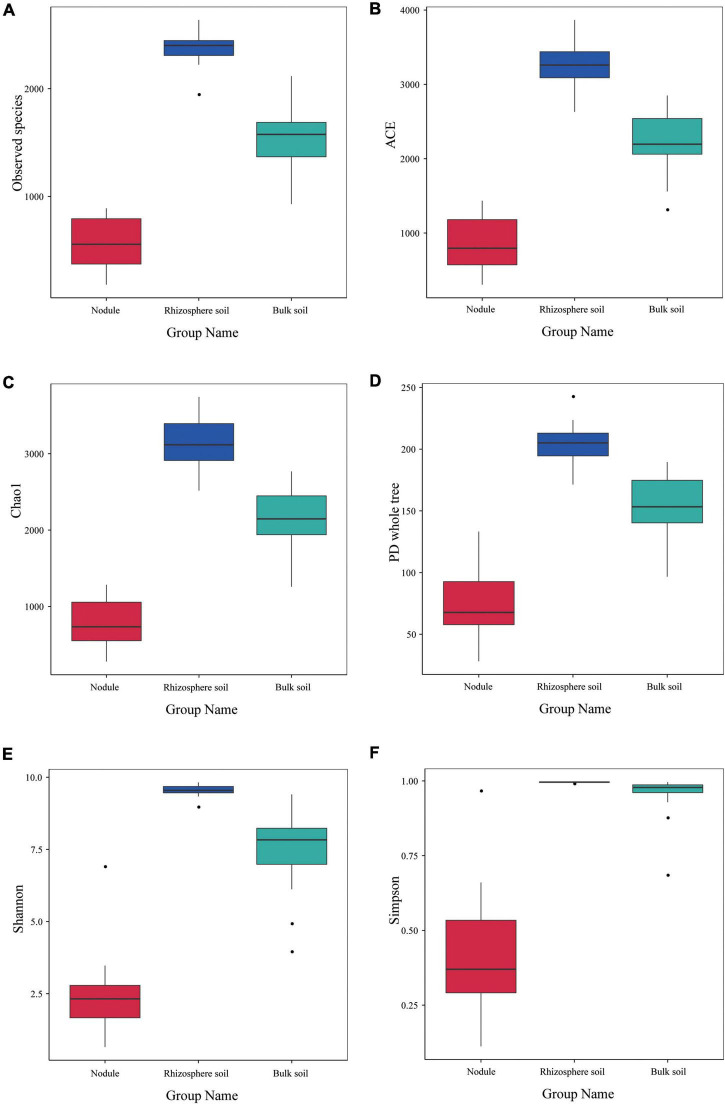
Alpha diversity index of the microbial communities within the three types of *S*. *davidii* samples. **(A–F)** represent observed species, and the results of the ACE, Chao1, PD Whole Tree, the Shannon and Simpson index of the different samples of nodules, rhizosphere soil and bulk soil. Statistical analyses were performed by the paired Wilcoxon rank-sum test and Tukey’s test. The significance is denoted by asterisks where ^⋅^*P* < 0.05.

The alpha diversity index of the three groups of samples is shown in Figure 5. The observed species index of the rhizosphere soil sample (2,383) was significantly higher than that of the bulk soil (1,544) and nodule sample (576; Figure 5A). The ACE, Chao1 and PD whole tree indices of the rhizosphere soil were 3,284.316, 3,152.988, and 204.567, respectively (Figures 5B–D). The ACE, Chao1, and PD whole tree indices of the root nodules were 867.9, 794.718, and 74.77 (Figures 5B–D), respectively. The ACE, Chao1 and PD whole tree indices in the bulk soil were 2,245.706, 2,152.737, and 151.514 (Figures 5B–D), respectively. The Shannon index and Simpson index of the rhizosphere soil were 9.533 and 0.995, respectively (Figures 5E,F). The Shannon and Simpson indices in the bulk soil were 7.498 and 0.954, respectively (Figures 5E,F). In the root nodules, Shannon and Simpson indices were only 2.434 and 0.405, respectively (Figures 5E,F). The above data showed that the community diversity and abundance within the rhizosphere soil were significantly higher than those of the bulk soil and the root nodules. The alpha diversity index indicates that the rhizosphere can recruit a variety of different microorganisms at the same time and has an enrichment effect on them. Nodules, on the other hand, act as filters, screening out a group of microbes that are beneficial to both themselves and their host plants.

### The rhizosphere microbial community is closely related to the composition of endophytic bacteria within the root nodules

**FIGURE 6:**
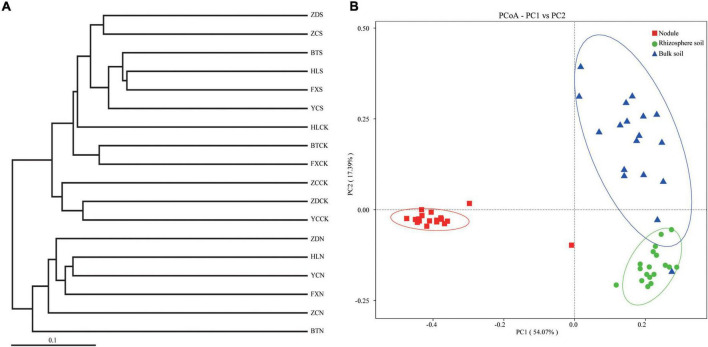
Beta diversity analysis. **(A)** is cluster pedigrees of different flora within the different samples based on unweighted UniFrac distances. **(B)** is principal coordinate analysis (PCoA) based on the relative abundance of bacterial OTUs.

Principal coordinate analysis, the UPGMA, and LDA effect size (LEfSe) analysis were used for beta diversity analysis to compare the microbial compositions of the different samples. The UPGMA tree showed that all the samples were divided into two different clusters: Group 1 was composed of root nodule samples, and Group 2 was composed of the rhizosphere soil and the bulk soil samples (Figure 6A). This indicates that the microflora in the root nodule is different from that in the rhizosphere soil. The PCoA results based on unweighted UniFrac distance showed that there were differences in microflora among different samples, which were 18.31% (PC1) and 7.78% (PC2), respectively (Figure 6B). The community structure of the rhizosphere soil was similar to that of the bulk soil, while there were significant differences in the bacterial community structure between the root nodules and the soil, indicating that the root nodules and the soil had their own unique bacterial community structure.

**FIGURE 7:**
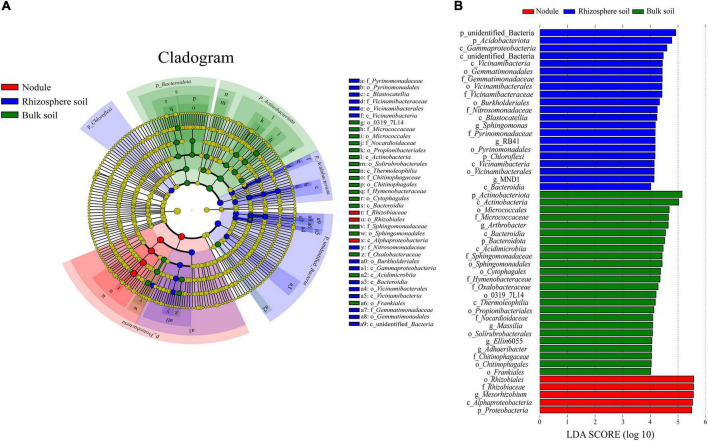
Results of the LEfSe analysis. **(A)** is phylogenetic cladistic diagram of the LEfSe. The circle of radiation from the center to the periphery represents the level of classification from the phylum to the species. Each small circle at different classification levels represents a classification at that level, and the diameter of the small circle is proportional to the relative abundance. **(B)** is histogram of the LDA value distribution (LDA > 4).

Through LEfSe analysis, we obtained species with significant differences in abundance in the different types of samples (Figure 7). At the family level (Figure 7A), Rhizobiaceae was significantly enriched within the root nodule samples. In the rhizosphere soil samples, Vicinamibacteraceae, Pyrinomonadaceae, Gemmatimonadaceae, and Nitrosomonadaceae were significantly enriched. Hymenobacteraceae, Chitinophagaceae, Nocardioidaceae, Micrococcaceae, Oxalobacteraceae, and Sphingomonadaceae were more abundant in the bulk soils. To assess the significance of discriminatively specific bacterial taxa, LEfSe analysis was accomplished from the phylum to genus across the three sites using the default logarithmic (LDA) value of 4 (Figure 7B). The clade graph showed that 24 bacterial groups were enriched in the bulk soil sample, and 21 were enriched in the rhizosphere soil sample, followed by the nodule sample (5 bacterial groups).

To further demonstrate the visual clustering of bacterial communities in the above analysis, different plant compartments were examined using ANOSIM analysis (Table 3). At the OTU level, the bacterial microbiota of all plant compartments differed significantly from one another.

**TABLE 3:** Analysis of similarity.

Phylogenetic level ANOSIM output	*R*	*P*
Root nodule VS rhizosphere soil	0.9777	0.001
Rhizosphere soil VS bulk soil	0.5469	0.001
Root nodule VS bulk soil	0.9925	0.001

0 ≤ R ≤ 1, indicated significant difference between sample groups. P < 0.05 indicated statistical significance.

### Bacterial communities associated with the soil’s physicochemical properties

**FIGURE 8:**
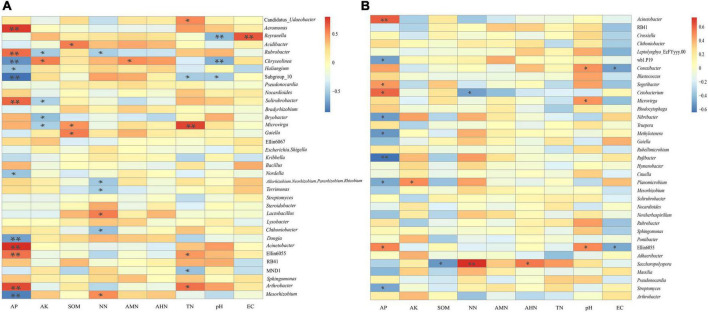
Heatmap of the correlation between the soil’s physicochemical properties and the genus-level bacteria. **(A)** is the correlation between physicochemical properties of rhizosphere soils and the genus-level bacteria. **(B)** is the correlation between physicochemical properties of bulk soils and the genus-level bacteria. * and ** indicate *P* < 0.05 and *P* < 0.01, respectively.

Table 4 shows that there were no significant correlations between the soil’s physicochemical properties and the bacterial diversity within the rhizosphere soil and the bulk soil. To further clarify the interactions between the soil’s physicochemical properties and the microorganisms, we analyzed the correlation between the top 35 species and the soil’s physicochemical properties at the genus level (Figure 8). In the rhizosphere soil samples, *Arthrobacter*, Ellin6055, *Acinetobacter*, *Solirubrobacter*, *Rubrobacter*, and *Aeromonas* possessed an extremely significant positive correlation with the AP. *Microvirga* was positively correlated with the TN. *Reyranella* was positively correlated with EC. *Mesorhizobium*, *Dongia*, Subgroup 10 and *Chryseolinea* possessed an extremely significant negative correlation with AP. *Chryseolinea* and *Reyranella* possessed an extremely significant negative correlation with pH. In the bulk soil samples, *Acinetobacter* and *Rufibacter* were significantly positively correlated and negatively correlated with AP, respectively. There was a very significant positive correlation between *Saccharopolyspora* and NN. In general, AP interacted with the bacterial diversity in both the rhizosphere soil and the bulk soil. TN had significant effects on some bacterial diversity within the rhizosphere soil but had no significant effects on the bacterial diversity within the bulk soil samples. AK, SOM, NN, pH, and EC were all related to bacterial diversity within the different types of soil.

**TABLE 4:** Correlations Analysis between bacterial alpha-diversity and soil physicochemical properties.

Environmental factors	Rhizosphere soil			Bulk soil		
	Observed_species	Chao1	Shannon	Observed_species	Chao1	Shannon
AP	0.383	0.484*	–0.200	–0.346	–0.420	–0.207
AK	–0.065	–0.154	0.195	–0.173	–0.160	–0.071
SOM	0.040	–0.026	0.230	0.057	–0.020	0.036
NN	–0.186	–0.195	0.010	0.385	0.245	0.434
AMN	–0.137	–0.329	0.259	0.373	0.261	0.257
AHN	–0.178	–0.288	0.031	0.249	0.144	0.313
TN	0.342	0.247	–0.095	0.044	–0.018	0.077
pH	0.130	0.147	–0.166	0.036	0.037	0.091
EC	–0.003	–0.030	0.136	–0.174	–0.191	–0.207

“*” indicate the significant correlations at 0.05 level. AP, available phosphorous; AK, available potassium; SOM, organic matter; NN, nitrate nitrogen; AMN, ammonium nitrogen; AHN, alkaline hydrolyzable nitrogen; TN, total nitrogen; and EC, electrical conductivity. n = 18.

### Rhizocompartmental bacterial community co-occurrence network analysis

**FIGURE 9:**
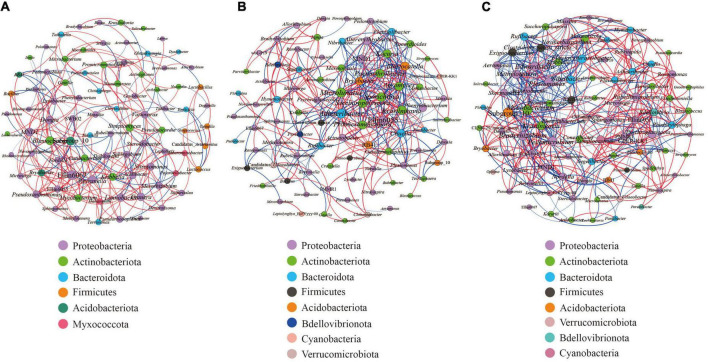
Microbial interaction networks in the different compartments. The larger the circle layer of the species is, the greater the connection between the circle layer is, and the higher the correlation degree with other species is, which represents the core species in the correlation. Different colors represent the taxonomic distribution of the corresponding species at the gate level. The edge color indicates a positive (red) and negative (blue) correlation. The width of the edge represents the relevant value. Only a significant correlations (*r* > 0.6 percent, *P* < 0.05) were shown. **(A)** is the co-occurrence network of the bacterial communities within the root nodule. **(B)** is the co-occurrence network of the bacterial communities within the rhizosphere soil. **(C)** is the co-occurrence network of bacterial communities within the bulk soil.

We carried out Spearman correlation analysis and statistical testing on the standardized 16S rRNA gene sequencing data, calculated the correlation between various species, selected the top 100 bacteria with the highest horizontal correlation, and drew the co-expression analysis network diagram based on gephi (Figure 9). Table 5 calculates the important topological properties of different samples to describe the complex patterns of the relationships between nodes. In the root nodules, the APL was 4,465, and the diameter was 14. The CC and modularity index (MD) were 0.410 and 0.576, respectively. In the rhizosphere soil, the APL was 3,767, and the diameter was 12. The CC was 0.599, and the MD was 0.380. In the bulk soil, the APL was 3,102, and the diameter was 11. The CC was 0.560, and the MD was 0.401. A MD value > 0.4 indicates that the network has a modular structure. It can be seen that the root nodule network diagram obviously has a modular structure. In addition, there were 280 correlations founds in the root nodule samples, 370 correlations in the rhizosphere soil samples and 555 correlations in the bulk soil samples. The interaction network of microorganisms in the root nodules is simpler than that in the soil, but the correlation between bacteria is enhanced. The network diagram showed that the bacterial community in root nodules was mainly composed of Proteobacteria, Actinobacteriota, Bacteroidota, Firmicutes, Acidobacteriota, and Myxococcota, in which Proteobacteria were dominant. At the genus level, Ellin6067, Subgroup 10, and *Blastococcus* showed a robust correlation with other genera (Figure 9A). Compared with root nodules, there were more dominant species of bacteria in the rhizosphere soil and the bulk soil. In the rhizosphere soil, *Adhaeribacter*, *Microlunatus* and Ellin6055 showed a wide range of robust correlations (Figure 9B). In the bulk soil, the composition of the bacterial community was the most complex at the phylum level, and there was a robust correlation among the Subgroup 10, *Edwardsiella*, *Bradyrhizobium*, and *Pedomicrobium* (Figure 9C).

**TABLE 5:** Topological properties of co-occurring networks.

Sample name	Modularity (MD)	Clustering coefficient (CC)	Average path length (APL)	Network diameter (ND)	Graph density (GD)	Average degree (AD)
Root nodule	0.576	0.410	4.465	14	0.039	7.776
Rhizosphere soil	0.380	0.599	3.767	12	0.049	9.796
Bulk soil	0.401	0.560	3.102	11	0.086	17.36

## Discussion

In recent decades, many studies have been focused on the bacteria residing in legume root nodules. In addition to rhizobia, other NREs have also been simultaneously isolated from the root nodules, including *Rahnella, Bacillus*, *Enterobacter*, *Pantoea*, *Agrobacterium*, *Pseudomonas*, *Cohnella*, *Herbaspirillum*, *Paenibacillus*, *Klebsiella*, *Endobacter*, *Chryseobacterium*, *Stenotrophomonas*, *Azospirillum, Gluconacetobacter, Variovorax*, *Arthrobacter*, *Micromonospora*, *Hyphomicrobium*, *Microbacterium*, *Rhodococcus*, *Frondihabitans, Kocuria*, *Providencia*, and *Sphingomonas*, from root nodules of several legume species (De Meyer et al., 2015; Martínez-Hidalgo and Hirsch, 2017; Rahal and Chekireb, 2021). These findings demonstrated that the existence of NREs in leguminous root nodules is a universal phenomenon, and the occurrence of certain rhizobia was correlated with the presence of a particular NRE, suggesting that their presence may not be accidental. Nevertheless, most studies are observational, and few focus on the co-habitation of diverse NREs inside nodules. Moreover, most of the previous studies have mainly involved analysis of the bacterial diversity of rhizobia and legume plant–microbe interactions. Little systemic observation has been paid to the non-symbiotic endophytic bacteria within leguminous root nodules, and their colonization meaning and ecological role are thus far unknown.

A recent study showed that other bacterial genera are present in *Trifolium* nodules commonly called NREs, and the data presented in this study suggest the presence of a large diversity of bacteria belonging to 50 genera within the nodules of indigenous legumes in Flanders (De Meyer et al., 2015). However, although a few novel non-rhizobia endophytes belonging to various genera have been isolated, the majority of the non-rhizobia bacterial community in the root nodules of several legume species remains uncharacterized. In a previous study, traditional techniques, such as microbial isolation and cultivation under laboratory conditions, were used to investigate the cultural endophytes within root nodules. However, only a small fraction of microorganisms (less than 1%) from the natural environment and root nodules are cultivable. To better understand the diversity of the bacterial microbiota in root nodules, more efficient technology is needed. In recent years, this problem has been resolved by next-generation sequencing of the 16S ribosomal RNA (rRNA) gene as a culture-independent molecular technique. Here, we have explored and characterized the diversity of NRE bacteria associated with *S*. *davidii*, a native N-fixing wild leguminous shrub that is a potential tool species for vegetation and ecosystem rehabilitation in hilly and gully regions of the Loess Plateau of China, by using 16S rRNA gene high-throughput amplicon sequencing, which allowed the full-depth analysis of bacterial community diversity within root nodules. In this study, we showed the presence of a large diversity of bacteria belonging to 81 phyla, 154 classes, 333 orders, 463 families, and 732 genera inside nodules associated with *S*. *davidii* of indigenous legumes. To our knowledge, this is the first report of such an extensive bacterial diversity analysis in root nodules of indigenous legumes in hilly and gully regions of the Loess Plateau of China, although similar study on cultured legumes in the same region have been reported (Xiao et al., 2017; Zai et al., 2021).

Legumes are known as pioneer plants and as enhancers of nutritional status in cultivated soils, which has been explained by their capacity to recruit rhizobia, since they give an indication of host specificity and nitrogen-fixing ability (Diouf et al., 2010). Legume root nodules constitute an environmental niche for the accommodation of specific traditional rhizobia and NRE bacteria. Previous studies found that *Mesorhizobium* sp. X, *M. waimense*, and *M. amorphae* were the dominant and universal microsymbionts of *S*. *davidii* in the forest area of the Loess Plateau in northern Shaanxi Province (Cao et al., 2021). However, to date, there has been no evaluation of the role of NREs in pioneer legumes of *S*. *davidii* grown in poor soils on the Loess Plateau of China. In the present study, we also found the dominant genus *Mesorhizobium* (75.07%) in the root nodules of *S*. *davidii*, which is consistent with the results reported by Cao et al. (2021), and confirmed that *Mesorhizobium* species were dominant symbionts for genus *Sophora* (Zhao et al., 2010; Jiao et al., 2015; Tan et al., 2015; Tagelsir and Rabab, 2017). Similarly, the legumes may select the specific and appropriate NRE from environment (De Meyer et al., 2015). Taken together with our findings, the leguminous plants not only select a certain and specific NRE but also the rhizobia inside the root nodules prefer certain NREs. Nevertheless, based on the above research background, the co-habitation of diverse bacteria inside nodules raises questions regarding the entry mode of endophytic bacteria and the role of the environment, since the biogeographic patterns and assembly process of rhizobial and NRE communities vary among root nodules, rhizosphere and bulk soil compartments. In an effort to address the issue mentioned above, we compared the biogeography and co-occurrence patterns of the bacterial communities obtained from root nodules samples, rhizosphere samples and bulk soil samples of *S*. *davidii* in hilly and gully regions of the Loess Plateau of China.

### Microbial composition difference in the root nodule, the rhizosphere, and the bulk soil

The physicochemical properties of the rhizosphere, which is surrounded and affected by plant roots, are different from those of the surrounding bulk soil (Dessaux et al., 2016). Previous studies have reported that the microbiomes of the rhizosphere are a part of the bulk soil community derived from root selection (Philippot et al., 2013; Mendes et al., 2014). However, the microbial community composition of the bulk soil and the rhizosphere environment compartments are distinctly different (Reinhold-Hurek et al., 2015), which revealed that its diversity decreases with proximity to the root (Bulgarelli et al., 2013; Donn et al., 2015). Based on alpha and beta diversity analyses, the bacterial richness and diversity from different habitats were compared, and we found that the microbial community composition of the rhizosphere soil differed from that of the bulk soil of *S*. *davidii*. Moreover, the alpha diversities of the microbial communities decreased from the bulk soil to the rhizosphere to the root nodule, which is consistent with the study of Xiao et al. (2017). Our results show that there were 63 phyla of endophytic bacteria within the root nodules, 69 phyla of bacteria within the rhizosphere soil, and 62 phyla of bacteria within the bulk soil. Similar to a previous study (Mendes et al., 2013), we observed in our survey that Proteobacteria was the most dominant phylum, and its proportion in the root nodules (85.77%) was much higher than that in the rhizosphere soil (28.93%). In the bulk soil, only Actinomycetes (32.61%) were enriched, suggesting that bacterial community distribution is habitat-specific (Shi et al., 2014).

Furthermore, an interesting gradient was observed in which the bacterial diversity decreased as the distance from the soil increased. In addition, the proportion of Acidobacteria in rhizosphere soil (12.71%) was significantly higher than that in the nodules (0.74%) and the bulk soil (3.76%), and this finding was consistent with previous reports in many plants and soils (Bulgarelli et al., 2012; Chen et al., 2019; Lin et al., 2019). In the present study, Proteobacteria were absolutely dominant within the root nodules, which may be the result of the differentiation of specific bacteria within different niches and the active filtration of bacteria by the host plants (Bulgarelli et al., 2013; Yuan et al., 2021). This might be because Proteobacteria thrive better under high nutrient conditions (Zhao et al., 2017) and tend to exploit more labile C sources (Fierer et al., 2007). Overall, these findings suggest that the alpha diversity with root proximity showed a decreasing trend owing to the root filtration and selection effect (Dennis et al., 2010). Additionally, previous studies have confirmed that Acidobacteria dynamics are affected by soil pH (Fan et al., 2017). The results of the soil’s physicochemical properties showed that all of the rhizosphere soil samples were alkaline, and the content of Acidobacteria was abundant, which indicated that the living environment of Acidobacteria was not limited to an acidic environment (Xiong et al., 2012).

### Important abiotic factors affecting microbial community structure

In this study, a total of 732 different genera were detected, and there were significant differences in the relative abundance of these bacteria. Comparison of the bacterial communities associated with *S*. *davidii* revealed both ubiquitous and specific members in different sampling sites, including *Sphingomonas*, *Mesorhizobium*, RB41 and *Arthrobacter*. Moreover, the bacterial richness and diversity varied in the three different compartments, with the highest bacterial diversity occurring in the rhizosphere soil. Similar results have been reported that plant endophytic bacteria mainly come from rhizosphere soil, and the microbial community within the rhizosphere soil can affect the bacterial diversity in or related to the root nodules (Leite et al., 2017). The bacterial community in the rhizosphere soil showed higher species richness than endophytic bacteria (Tan et al., 2017; Yang et al., 2017).

The pivotal influential factors and their contributions to variations in community composition were determined by PCoA (Figure 6). Our findings showed that microbial communities in the three compartments were associated with various edaphic physical and chemical properties, indicating that they had significantly different biogeographic patterns among the three compartments and that they had their own unique bacterial community structure. It is understood that most root endophytic bacteria are from the soil, and the rhizospheric microbiome is derived from the soil (Lennon and Jones, 2011; Philippot et al., 2013). Furthermore, Spearman’s correlation was used to test the effect of the soil’s physical and chemical properties on the relative abundances of the top 35 bacterial genera. The results showed that AP was significantly positively correlated with 6 bacteria (*Arthrobacter*, Ellin6055, *Acinetobacter*, *Solirubrobacter*, *Rubrobacter*, and *Aeromonas*) and negatively correlated with 4 bacteria (*Mesorhizobium*, *Dongia*, Subgroup 10, and *Chryseolinea*) in the rhizosphere soil samples (Figure 8), suggesting that bacterial communities and composition within the three different compartments were significantly related to the different soil properties. It is well known that soil P availability affects and influences the microbial community in roots and soil (Ren et al., 2021). Some reports have shown that soil AP is significantly relevant to the bacterial community of *S*. *davidii*, indicating that AP may be an important factor affecting the diversity and composition of the bacterial community in the rhizosphere (Pande et al., 2017; Zhou et al., 2020). It has been found that soil microorganisms can transform the combined phosphorus in soil into water-soluble phosphorus, thus promoting the absorption of phosphorus by plants (He and Zhu, 1998) and shifting the rhizosphere bacterial microbiome (Lennon and Jones, 2011). Soil nitrogen plays an important role in the biological nitrogen fixation of legumes (Taylor and Menge, 2018). In this study, a significant Spearman relationship was observed in which the TN, EC, pH and NN were also important factors correlating with the bacterial community variation (Figure 8).

All of these findings support the conclusion that the highest bacterial diversity observed in the rhizosphere soil may be attributed to the primary site of interaction between plants and soil, and plants play an important role in determining the community composition and structure of the rhizospheric microbiome by recruiting specific microbiomes (Berendsen et al., 2012).

### The co-occurrence patterns of microbes in the root nodule, the rhizosphere soil, and the bulk soil

Previous studies found that microbial networks were more complex within bulk soil than in the rhizosphere (Mendes et al., 2014; Fan et al., 2017). In the present study, network analysis of *S*. *davidii*-associated microbial communities within the root nodule, the rhizosphere and the bulk soil in hilly and gully regions of the Loess Plateau of China was conducted. Our results revealed that the microbial network diagram of the co-occurrence pattern showed that more complexes were observed in soil than in the root nodules, namely, the links and nodes decreased from the bulk soil to the rhizosphere soil to the root nodules (Figure 9), which demonstrated that the network complexity of the bacterial communities in the three different compartments is consistent with the above result of diversity. The rhizosphere community is generally considered a subset of the bulk soil community. This further proves that the host plants will actively filter bacteria (Bulgarelli et al., 2013), leaving useful bacterial interactions to promote the growth and development of host plants. These findings indicated that only specific microorganisms could colonize and thrive in the rhizosphere through the selection and filtering effects of roots.

Moreover, the microbial network in the root nodules showed more positive correlations. Thus, the NREs in the root nodules may have a higher nutritional dependence on rhizobia, resulting in a higher proportion of NRE-rhizobial edges in the root nodule networks, further indicating that the coexistence of the rhizobia and certain NRE bacteria in the root nodule was cooperative and had beneficial effects on the host plants. Taken together, these results showed that the networks in root nodules had more rhizobia and certain NRE bacterial links than the networks in both the rhizosphere and the bulk soil.

## Conclusion

Our results demonstrated that the microbial community composition was distinctly different in root nodules and the rhizosphere and bulk soil environment compartments of *S*. *davidii* in hilly and gully regions of the Loess Plateau of China, indicating that the environmental filtering differed in the assembly of bacterial communities. Soil AP influenced the bacterial communities in the bulk soil, suggesting that there were significantly different biogeographic patterns among the three compartments and that they had their own unique bacterial community structure. The networks in the root nodules had more rhizobia and certain NRE bacterial links than the networks in both the rhizosphere and the bulk soil samples. These findings indicated that only specific microorganisms could colonize and thrive in the rhizosphere through the selection and filtering effects of roots. Overall, our study improves the comprehension of the biogeographic patterns and diversity of bacterial microbiota inhabiting root nodules and can help elucidate the root nodule assemblage process of *S*. *davidii*.

## Data availability statement

The data presented in the study are deposited in the GenBank Data repository, accession number PRJNA851563.

## Author contributions

LJ, JY-Y, ZC-C, ZR-H, and AJ-M performed the material preparation, experimental operation, data collection, and analysis. LJ and DZ-S wrote the manuscript. LJ and LX-D performed the project guidance and critical revision of manuscripts. All authors read and approved the final manuscript.

## Conflict of interest

The authors declare that the research was conducted in the absence of any commercial or financial relationships that could be construed as a potential conflict of interest.

## Publisher’s note

All claims expressed in this article are solely those of the authors and do not necessarily represent those of their affiliated organizations, or those of the publisher, the editors and the reviewers. Any product that may be evaluated in this article, or claim that may be made by its manufacturer, is not guaranteed or endorsed by the publisher.

## Footnotes

1
http://ccb.jhu.edu/software/FLASH/

2
http://qiime.org/scripts/split_libraries_fastq.html

3
https://www.arb-silva.de/

4
http://www.drive5.com/usearch/manual/uchime_algo.html

5
http://drive5.com/uparse/

6
http://www.drive5.com/muscle/
