# Supplementary material for: Environmental filtering drives the establishment of the distinctive rhizosphere, bulk, and root nodule bacterial communities of Sophora davidii in hilly and gully regions of the Loess Plateau of China
Source: Front Microbiol. 2022 Jul 22;13:945127. doi: 10.3389/fmicb.2022.945127 (PMC9355530; doi:10.3389/fmicb.2022.945127)
Supplement: Supplementary file 1 [file Table_2.DOCX]

Supplementary material table 1. Location of the study plots

| Site | | Annual average temperature (◦C) | Annual average precipitation (mm) | Altitude | Location of site (GPS) |
| --- | --- | --- | --- | --- | --- |
| Huangling | Plot 1 | 9.4 | 568.8 | 765 m | 35^◦^34’20.05”N, 109^◦^23’18.52”E |
|  | Plot 2 |  |  | 864 m | 35^◦^34’49.58”N, 109^◦^15’16.3”E |
|  | Plot 3 |  |  | 867 m | 35^◦^38’27.42”N, 109^◦0^4’49.28”E |
| Fuxian | Plot 1 | 9.8 | 550.3 | 1011 m | 36^◦^00’1.17”N, 109^◦^25’59.65”E |
|  | Plot 2 |  |  | 893 m | 35^◦^58’14.26”N, 109^◦^22’17.59”E |
|  | Plot 3 |  |  | 908 m | 36^◦^01’44.82”N, 109^◦^23’53.41”E |
| Baota | Plot 1 | 7.0 | 550.0 | 973 m | 36^◦^37’24.76”N, 109^◦^27’23.83”E |
|  | Plot 2 |  |  | 1085 m | 36^◦^27’47.1”N, 109^◦^26’36.47”E |
|  | Plot 3 |  |  | 972 m | 36^◦^41’42.04”N, 109^◦^41’22.98”E |
| Yanchang | Plot 1 | 10.4 | 564.0 | 836 m | 36^◦^35’36.2”N, 109^◦^53’49.24”E |
|  | Plot 2 |  |  | 833 m | 36^◦^32’58.77”N, 110^◦^4’15.57”E |
|  | Plot 3 |  |  | 816 m | 36^◦^31’14.3”N, 110^◦^12’0.05”E |
| Zhidan | Plot 1 | 8.1 | 474.2 | 1200 m | 36^◦^45’47.95”N, 108^◦^46’31.16”E |
|  | Plot 2 |  |  | 1258 m | 36^◦^51’36.86”N, 108^◦^52’30.51”E |
|  | Plot 3 |  |  | 1458 m | 36^◦^54’36.93”N, 108^◦^48’0.81”E |
| Zichang | Plot 1 | 9.1 | 514.7 | 1244 m | 37^◦^5’8.11”N, 109^◦^28’5.92”E |
|  | Plot 2 |  |  | 1244 m | 37^◦^4’40.99”N, 109^◦^33’8.58”E |
|  | Plot 3 |  |  | 1186 m | 37^◦^7’50.43”N, 109^◦^39’3.58”E |
